# Noshing on Chocolate, I Can Do That: Increased Chocolate Consumption in the Chocolate‐Modified Bogus Taste Test With Better and Not Worse Inhibitory Control

**DOI:** 10.1002/erv.3206

**Published:** 2025-05-19

**Authors:** Philipp A. Schroeder, Anton Ernst, Robert Wirth, Nils B. Kroemer, Jennifer Svaldi

**Affiliations:** ^1^ Department of Psychology Clinical Psychology & Psychotherapy University of Tübingen Tübingen Germany; ^2^ German Center for Mental Health (DZPG) Partner Site Tübingen Germany; ^3^ Department of Psychology University of Würzburg Würzburg Germany; ^4^ Section of Medical Psychology Department of Psychiatry and Psychotherapy Faculty of Medicine University of Bonn Bonn Germany; ^5^ Department of Psychiatry and Psychotherapy Tübingen Center for Mental Health University of Tübingen Tübingen Germany; ^6^ German Center for Diabetes Research (DZD) Neuherberg Germany

**Keywords:** bogus taste test, chocolate, inhibitory control, virtual reality

## Abstract

**Background:**

Chocolate is the most craved energy‐dense food. Yet, most individuals can limit their chocolate consumption. Here, we investigate the cognitive mechanisms underlying chocolate consumption in a chocolate bogus taste test in a cross‐sectional experimental design.

**Method:**

High chocolate cravers abstained from chocolate for a week, followed by a virtual reality chocolate exposure with biometric trajectory recordings of their stopping responses and an ad‐libitum bogus taste test of spontaneous chocolate intake. A single‐target implicit association task and a computerised stop‐signal task served as unstandardised control tasks 1–2 days before chocolate intake.

**Results:**

Associations of parameters from all tasks with chocolate intake were small (|*r*| < 0.23). Elastic net models misestimated food intake by min. 160 kcal (generalisation: 180 kcal) and feature selection was only possible with L1 penalty. At the group level, participants showed a more controlled and delayed movement towards chocolate relative to neutral cues, evidenced by lower peak acceleration and peak velocity and faster stopping latency.

**Discussion:**

The findings demonstrate the complex cognitive‐behavioural underpinnings of food intake, food craving and abstinence.

**Summary**

- Inhibitory control for chocolate in trait chocolate cravers is enhanced in biometric measures derived from virtual reality trajectory recordings.
- Feature selection models indicate trait craving, impulsivity, stop‐signal reaction time, implicit avoidance, peak velocity and total dwell time as concurrent predictors of chocolate intake.
- A small negative correlation (*r* = −0.21) suggested higher spontaneous chocolate intake in chocolate cravers with superior inhibitory control abilities.

## 1 Introduction

Chocolate and chocolate‐containing foods account for approximately half of all food craving episodes (Bruinsma and Taren 1999; Hill 2007; Richard et al. 2017), particularly among females (Hormes et al. 2014). Chocolate cravings typically correspond with subsequent chocolate intake (Meule and Hormes 2015). At the same time, chocolate snacks are often energy‐dense and readily available, at least in obesogenic Western civilisations prone to overeating, overweight and obesity. Not all humans overeat, but enormous prevalence rates of up to 33% of obesity with a BMI of 30 kg/m^2^ and greater are considered an obesity pandemic with detrimental consequences on individual health (particularly cardiovascular diseases) (Emerging Risk Factors Collaboration et al. 2011; Y. Wang et al. 2020; World Health Organization 2020; Wyatt et al. 2006). Chocolate snacking, furthermore, often constitutes a merely hedonic intermediate meal that is not necessarily linked to physiological hunger (Cleobury and Tapper 2014; Meule and Hormes 2015). In elucidating the cognitive mechanisms underlying food craving and spontaneous (over‐)eating, chocolate‐related behaviour is therefore a unique and informative research area.

Inhibitory control is the ability to suppress prepotent motor responses (Diamond 2013; Logan et al. 1984; Verbruggen et al. 2019), and deficits in inhibitory control have been described repeatedly in individuals with overweight and obesity (Bartholdy et al. 2016; Lavagnino et al. 2016; Svaldi et al. 2015; Wu et al. 2013). Inhibitory control refers to the process of suppressing thoughts and actions (early‐stage) or cancelling an initiated motor response (late‐stage). Several studies have described a negative association between higher BMI and worsened late‐stage inhibitory control, often conceptualised in the stop‐signal reaction time (SSRT; Houben et al. 2014; Schroeder et al. 2021). Late‐stage inhibitory control is typically measured in the stop‐signal task, a behavioural paradigm that requires participants to press a key upon the presentation of a stimulus in most trials of the task, but to interrupt their response in a subset of stop‐trials (Logan et al. 1984; Verbruggen et al. 2019). SSRT is the estimate of inhibitory control derived from the task and describes the latency of the stop process. However, due to the perplexing requirement of the task to observe and describe a non‐existing behaviour, that is, the non‐execution of a response, the estimate of SSRT is measured *indirectly*. More precisely, SSRT assumes a horse‐race between the go‐ and the stop‐process, which enables researchers to estimate stopping latencies from the systematic variation of the stop‐signal delay (the delay between stimulus onset and stop‐signal onset) and the go response latencies.

Recently, several proposals were made to measure inhibitory control *directly.* Continuous recordings of muscle activity in electromyography, for example, could differentiate go‐ and stop‐trials and characterise the stopping process (Raud et al. 2020) similar to response force recordings (Z. Wang et al. 2024). Both approaches circumvent the horse race model by relying on subthreshold muscle activity prior to a motor response and quantifying complete stopping. Alternatively, mouse tracking approaches have been proposed that provide additional biometric parameters, for example, mouse velocity (Benedetti et al. 2020; Bravi et al. 2022) and the spatial displacement of the mouse cursor following the stop‐signal presentation (Leontyev and Yamauchi 2019). Similar to this approach, Schroeder and colleagues recently introduced a stopping task in virtual reality (VR) that records participants' hand trajectories in go‐ and stop‐trials (Schroeder et al. 2023, 2024). In both mouse tracking and VR scenarios, a motor action is already initiated before the stop‐signal is presented and the ongoing action trajectory reveals the process of stopping directly in continuous spatial and temporal recordings of the mouse or controller position.

The VR stopping task is expected to measure action cancelation to immersive stimuli more specifically, and to provide measures of late‐stage inhibitory control in temporal data (i.e., similar to the indirect estimation of SSRT) and in spatial data (i.e., distance after a stop‐signal was shown). Notably, spatio‐temporal measures from VR were only weakly correlated with SSRT, but more strongly correlated with chocolate craving and impulsivity (Schroeder et al. 2023).

Regarding the clinical validity of direct measures of inhibitory control, Leontyev and Yamauchi (2019) reported a stronger association of mouse trajectories with self‐reported impulsivity compared to SSRT. Moreover, their prediction of subclinical symptoms of attention‐deficit hyperactivity disorder improved by including mouse trajectories in a feature selection model. In VR, Schroeder et al. (2024) were previously able to differentiate healthy controls from patients with anorexia nervosa in trajectory responses to high‐calorie food (Schroeder et al. 2024). However, none of the direct recordings of inhibitory control had been evaluated regarding their predictive validity regarding food intake. To the contrary, a very recent meta‐analysis of SSRT with 16 studies could only show a small positive association (*r* = 0.15) with food intake/food choice (McGreen et al. 2023).

In VR stopping scenarios, participants interact with immersive food‐related cues. As an advantage, the task enables researchers to investigate late‐stage stopping reactions directed to such cues without having to vary the stop‐signal delay across many blocks of trials. Virtual food in VR has been shown to influence food choice and consumption (Nederkoorn et al. 2009), to elicit food cravings in general (Ferrer‐Garcia et al. 2017; Ledoux et al. 2013) and to elicit chocolate craving specifically (Schroeder et al. 2023; van der Waal et al. 2021). Given the high sensory qualities of food stimuli, it is possible that VR‐based paradigms might reflect associations between inhibitory control and food intake more accurately. To address this research question in the present research, we conducted a study measuring chocolate consumption in the lab in a chocolate‐modified bogus taste test. The bogus taste test is an ecologically valid laboratory measurement that indicates the spontaneous food intake (Hallschmid et al. 2012; Robinson et al. 2017; Vöhringer, Hütter, et al. 2023). It is a measure sensitive to experimental manipulations such as craving induction (Larsen et al. 2012), food cue exposure (Jansen et al. 2011) or mood induction (Cardi et al. 2015; Svaldi et al. 2014). Previous SSRT studies showed lower food intake with better inhibitory control (Allom and Mullan 2014; Giesen et al. 2012; Guerrieri et al. 2008; Guerrieri, Nederkoorn, and Jansen 2007; Guerrieri, Nederkoorn, Stankiewicz, et al. 2007; Hofmann et al. 2009; Houben et al. 2012; Nederkoorn et al. 2009, 2015; van Strien et al. 2014).^1^

Accordingly, the present study tested the association between VR inhibitory control to chocolate stimuli and chocolate intake. We invited normal‐weight females with high chocolate cravings after a self‐governed chocolate abstinence of 1 week. This was expected to increase chocolate cravings and to maximise chocolate intake in the lab. In a cross‐sectional design, we recorded biometric stopping trajectories in a VR task in response to virtual chocolate and neutral objects (shoes), after which their spontaneous chocolate consumption was recorded in the chocolate taste test. The VR task included two stimulus categories to test chocolate‐specific inhibitory control (Schroeder et al. 2023) and eye‐tracking to measure visual attention (Hardman et al. 2021; Werle et al. 2024; Werthmann et al. 2013). Before testing, two web‐based control tasks measured SSRT in an adaptive stop‐signal task (Verbruggen et al. 2019) and chocolate approach tendencies in a single‐category implicit association test (Kemps et al. 2013). We were primarily interested in the predictive validity of the biometric data and expected higher calorie intake with longer attention (via eye‐tracking in VR), farther approach (via spatial parameters of the hand movements) and longer stopping latencies (via temporal aspects of the hand movements) to chocolate objects. Similarly, we expected higher calorie intake with higher implicit chocolate‐approach and worse inhibitory control in the web‐based tasks, but less pronounced. Both control tasks were conducted autonomously during the fasting period to shorten contact times in the lab during the ongoing COVID‐19 pandemic and thus should provide relatively conservative (i.e., underestimated) associations with food intake, relative to the measures from VR. Since we expected some redundancies in the exploratory power of our predictors, we further hypothesised that a feature selection machine learning algorithm would indicate the best predictor of chocolate intake out of a variety of established and novel biometric parameters.

## 2 Methods

The study and its main aims were preregistered at the Open Science Framework (anonymised link: https://osf.io/chwpf/). We performed and reported additional analyses for feature selection (i.e., elastic net regression) after initial data analysis.

### 2.1 Participants

We recorded biometric data from *N* = 72 female chocolate‐abstaining chocolate cravers (mean age = 22.9 years, SD = 3.73; see Table 1). Exclusion criteria were male biological sex, left‐handedness (with a right‐handedness score of 50 or less (Oldfield 1971)), pregnancy or current lactation, allergies (e.g., to nuts), BMI < 18.5 kg/m^2^ or BMI > 29.9 kg/m^2^, age < 18 years or age > 35 years. This study was approved by the local ethics committee (Az: Schröder 2019_0919_166). All participants were informed about the study procedures, signed informed consent, and were remunerated financially or through course credit. We used authorised deception for the bogus taste test and all participants were informed about the recording of their chocolate intake after termination of the study.

**TABLE 1:** Demographic characteristics of participants, mean age (SD) and mean questionnaire scores (SD).

	Mean (SD)	Minimum	Maximum
Mean age	22.85 (3.73)	18	34
Mean BMI (kg/m^2^)	22.55 (2.66)	18.7	29.8
Trait chocolate craving [FCQ‐T‐r‐Ch]	58.74 (9.93)	38	76
State chocolate craving [FCQ‐S‐r‐Ch]	51.21 (12.18)	19	77
Impulsivity [BIS‐15]	25.20 (5.90)	15	19
EDE‐Q	1.27 (0.89)	0.09	4.32
PHQ‐9	6.75 (3.22)	0	14

*Note:* The mean values in all groups were below the screening cut‐off for eating disorders and depression.

Abbreviations: BMI = body mass index, EDE‐Q = eating disorder examination – questionnaire, FCQ = food craving questionnaire (trait and state chocolate version), PHQ‐9 = patient health questionnaire 9.

### 2.2 Definition of Chocolate Craving

Based on a pilot study of 165 healthy females who were recruited in the same mid‐sized town, a median score was determined for trait chocolate craving using the Food Craving Questionnaire‐Trait‐revised Chocolate Version (FCQ‐T‐r‐Ch) (Meule and Hormes 2015). The FCQ‐T‐r‐Ch contained 15 items on chocolate craving, for example, ‘*I find myself preoccupied with chocolate*’ or ‘*If I give in to a food craving, all control is lost*’. All items were rated on a 6‐point scale from *never* to *always*. In the pilot study, the median score was *M* = 37, with an excellent internal validity of the questionnaire (Cronbach’s *α* = 0.94). We here used the median as threshold for the recruitment of chocolate cravers.

### 2.3 Procedure

Participants were invited to the study following a brief web‐based survey. We limited the sample on females who crave more frequently, intensely, and who also reported more eating‐related psychopathology than men (Hormes et al. 2014). Only females with chocolate food cravings above threshold (FCQ‐T‐r‐Ch score > 37) were included. Before testing, participants agreed to abstain from any chocolate consumption for 1 week, a deprivation period that previously increased chocolate wanting, liking, and consumption (Blechert et al. 2014). They received daily reminders and diary links. We recorded two conventional measures of inhibitory control (web‐based stop‐signal task) and of implicit chocolate approach (web‐based single‐category implicit association test) within 48 h before testing (see Supporting Information S1 for details). After arrival at the laboratory, the VR task (45 min) and the bogus taste test (BTT, 20 min) were conducted.

### 2.4 VR Task

**FIGURE 1:**
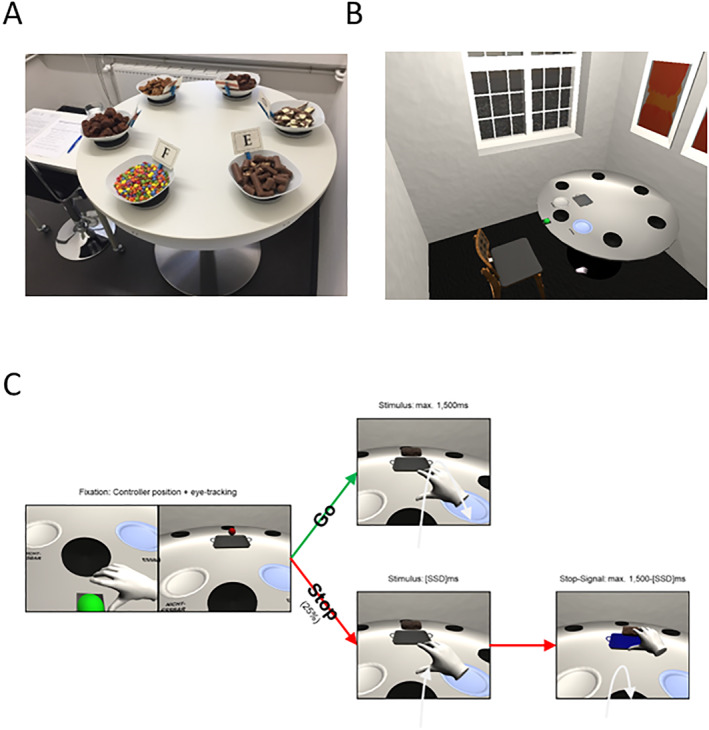
Arrangements of the chocolate implementation of the bogus taste test (A) and depiction of the VR environment (B), that was a virtual version of the same room. (C) Shows example go‐ and stop‐trials of the VR task.

Participants were equipped with a HTC‐Vive head‐mounted display (HTC Corporation, Taoyuan, Taiwan) with a built‐in near infrared eye‐tracking plug‐in by SMI (SensoMotoric Instruments, Teltow, Germany) and a single 6‐degrees‐of‐freedom HTC Vive Wand controller for the tracking of their right hand. Details of the task were reported in the supplementary methods (see also Schroeder et al. 2023). In brief, participants were placed in a virtual instance of the snacking room for the subsequent bogus taste test (Figure 1). They were then confronted in 400 trials with virtual instances of the chocolate food or with neutral stimuli. Their task was to pick up the target object with their right hand and place it onto one of two plates (at the left/right sides of the starting position), dependent on whether the target object depicted chocolate or a shoe. However, in 25% of the trials, a stop‐signal was shown after movement onset (i.e., after participants had initiated their movement; dynamic starting line (Scherbaum and Kieslich 2018)). In stop‐trials, participants were required to cancel their hand movement and to stop as quickly as possible.

### 2.5 Bogus Taste Test

Spontaneous chocolate intake was measured naturalistically in a bogus taste test, followed by an established and extended procedure (Hallschmid et al. 2012; Vöhringer, Hütter, et al. 2023; Vöhringer, Schroeder, et al. 2023) by using six prearranged chocolate snacks (see Figure 1). The chocolate snacks were small double‐chocolate muffins (424 kcal/100 g), mini chocolate cookies (502 kcal/100 g), chocolate brownies (473 kcal/100 g), milk and white chocolate bar (530 kcal/100 g), milk, caramel and biscuit chocolate bar (493 kcal/100 g) and sugar‐coated chocolate bonbons (478 kcal/100 g). Muffins, brownies and chocolate bars were broken into random size smaller pieces. Then, each snack was filled in a large white bowl to an equal volume (approximately 2/3 filled), so the participants did not restrict their consumption due to possible observation of their eating behaviour. After a detailed instruction, participants were left alone for 20 min to taste and rate every snack answering 9 visual analogue scales each, and to provide a ranking of all chocolate snacks on chocolate‐related perceptual dimensions such as sweetness and crunch. It was emphasised that they could eat as much as they wanted while completing the ratings. Each bowl was secretly weighed before and after the taste test.

### 2.6 Questionnaires

Details and internal consistency of the employed questionnaires are reported in the supplement. For prediction, we measured the trait and state chocolate versions of the food craving questionnaire (FCQ‐T‐r‐Ch/FCQ‐S‐r‐Ch) (Meule and Hormes 2015) and the Barratt Impulsiveness Scale (BIS‐15) as a validated 15‐item short‐version of trait impulsivity (Meule et al. 2011).

### 2.7 Data Preprocessing

#### 2.7.1 Virtual Reality Task

Biometric trajectories and eye‐tracking in VR were recorded at 120 Hz. For preprocessing, we used the mousetrap()‐package for visualisation of full trajectories (Kieslich and Henninger 2017) and custom MATLAB script for extraction of parameters (see Wirth et al. 2020 for an overview). Trials were segmented from stimulus onset to reaching the maximal depth displacement to identify parameters in the first movement phase (i.e., before grasping or stopping to targets). Trajectories were centred to the exact starting position in frame 1 (0/0/0). Predictor variables from trajectories were extracted from the continuous recordings (see Table 2). We used an outlier filter of 2.5 SD for all variables before averaging to remove excessive values in each participant and condition.

**TABLE 2:** List of predictor variables gathered in the web‐based experimental tasks and the biometric data gathered from virtual reality.

Abbreviation	Parameter [unit]	Description (*Interpretation*)	Task & trial type
SSRT	Stop signal reaction time [ms]	Indirect estimated stopping latency;	Stop‐signal task
		Estimated according to the integration method with replacement of response omissions (approximately SSRT = RT–SSD)	
		*Inhibition*	
RT	Reaction time [ms]	Time for completion of trial	Stop‐signal task: Go
		*Response efficiency*	
SSD	Stop signal delay [ms]	Time until stop signal was displayed	Stop‐signal task: Stop
		*Inhibition*	
FA	False alarms [% y/n]	Indicates if stop‐trial was solved successful	Stop‐signal task: Stop
		Due to the tracking algorithm should return a stop probability of ∼0.5 (between 0.25 and 0.75, for calculation of SSRT)	
		*Inhibition*	
D‐score	Difference score and implicit association test effect [standardized]	Standardized difference score between the compatible and incompatible test block, i.e. approach + chocolate versus avoidance + chocolate. *Implicit chocolate approach*	Single‐category implicit association test
PV	Peak velocity [m/s]	Maximum speed of hand movement	VR: Go
		*Approach*	
PA	Peak acceleration [m/s²]	Maximum change in speed of hand movement	VR: Go
		*Approach*	
TTPV	Time to peak velocity [ms]	Time until maximum speed	VR: Go
		*Approach*	
TTPA	Time to peak acceleration [ms]	Time until maximum acceleration	VR: Go
		*Approach*	
TTS	Time to stop [ms]	Time after display of stop‐signal until maximum spatial displacement of hand towards stimulus	VR: Stop
		*Inhibition*	
BD	Breaking distance [m]	Length of movement from stop‐signal onset until maximum spatial displacement of hand towards stimulus	VR: Stop
		*Inhibition*	
maxZ	Maximum spatial displacement [m]	Absolute length of movement towards stimulus	VR: Stop
		*Relative Inhibition*	
ACC	Accuracy [y/n]	Indicates if stop‐trial was solved successful	VR: Stop
		Due to the tracking algorithm should return a stop probability of ∼0.5 (between 0.25 and 0.75, for calculation of SSRT)	
		*Manipulation check*	
IT	Initiation time [ms]	Time after stimulus presentation until hand movement is initiated	VR: Go + stop
		Note: Stop signal is always displayed after initiation time + SSD	
		*Attention*	
SSD	Stop signal delay [ms]	Time until stop signal was displayed	Stop
		*Inhibition*	
RT	Reaction time [ms]	Time for completion of trial	Go
		*Response efficiency*	
Dwell_1	First dwell time [ms]	Duration of first stimulus fixation (until detachment is detected)	Go & stop
		*Early Attention*	
Dwell_total	Total dwell time [ms]	Total duration of stimulus fixation (until end of trial)	Go
		*Attention*	

With spatial displacement, we refer to the most excessive hand position that was extracted from each trial's segmented data. Because the *x*_1_–dimension would reflect approach movements in this experiment, we hypothesised spatial displacement in the VR environment *x*_1_–axis (depth) to reflect successful stopping in stop‐trials. As the starting position was slightly lower than the target position, we also explored spatial displacement in the *x*_3_–axis (height).

The time to stop a previously initiated movement was determined from correct stop trials. In the trajectories, the maximal spatial displacement (see above) was first determined. We then calculated the time difference between stop‐signal onset and the timestamp for this position as the direct stopping latency.

Estimates of peak acceleration and peak velocity were extracted from individual trajectories. We used both unstandardised trajectories to obtain raw kinematic parameters and time‐standardized trajectories for comparable movement duration. According to our hypothesis, we explored stimulus differences between chocolate and control stimuli in go‐trials.

Eye‐tracking was recorded together with biometric data in the Unity game engine and was resampled online to 120 Hz. We used custom scripts within the game engine to detect collisions between gaze vectors and predefined areas of interest, that is, the fixation sphere and the stimuli. Within the script, the initial dwell time (from stimulus onset until first gaze detachment) and the total dwell time (including initial dwell time and all following fixations of the stimulus) was processed for each trial.

We deviated from our preregistration and abstained from an estimation of SSRT as an indirect measure of inhibitory control in the VR task. This was decided because the requirements of SSRT estimation were not fulfiled in a majority of participants (i.e., stopping probability was above threshold). As reported in our results, due to the adaptive starting procedure and despite the adaptive stop‐signal delay, participants were much more successful in stopping in VR compared to the web‐based stop‐signal tasks. Instead, several direct measures of inhibitory control were extracted from their movement trajectories and their derivates.

### 2.8 Statistical Analysis

We reported Pearson product‐moment correlations for observations of predictive validity from the tasks. Correlation coefficients are interpreted as effect sizes according to Cohen (1988), that is: *r* > 0.1 is a small effect size, *r* < 0.3 is a medium‐sized effect size and *r* > 0.5 is a large effect size.

The web‐based stop‐signal task and the VR stopping task also manipulated the stimulus category (chocolate vs. neutral) within‐participants. In the VR stopping task, the trial‐type (stop‐trial vs. go‐trial) was additionally available as within‐participants manipulation. For biometric parameters, we expected the differences between stop‐trials and go‐trials to indicate the validity of the task in a manipulation check. We also explored possible stimulus‐category differences to replicate our previous findings of superior inhibitory control to chocolate (Schroeder et al. 2023). However, for the estimation of predictive validity, we also predefined whether measures were gathered from stop‐trials (e.g., to indicate stopping latency or stopping distance) and we were primarily interested in the data from trials with chocolate stimuli, since those trials were thought to better predict chocolate intake. Data from trials with neutral stimuli were also analysed and reported in the same way.

For feature selection, we constructed several models that predicted chocolate intake from the various parameters collected in the tasks.

Analyses were conducted using R (R Core Team 2020) together with the *tidyverse* (Wickham et al. 2019), *schoRsch* (Pfister and Janczyk 2016), *implicitMeasures* (Epifania et al. 2020)*, ez* (Lawrence 2016) and *rstatix* (Kassambara 2021). Elastic net and Lasso regression were implemented in *Google Colab*. We used a standard statistical threshold for testing of statistical significance (*α* = 0.05).

## 3 Results

### 3.1 Preliminary Analyses

Chocolate consumption in the bogus taste test ranged from 171–1141 kcal (*M* = 529 kcal, SD = 230 kcal). Participants reported moderate state craving for chocolate before chocolate intake (FCQ‐S: *M* = 52.7, SD = 13.6, range: 15–79), and before running the web‐based tasks (VAS: *M* = 57.7, SD = 27.6, range: 2–100). Chocolate craving did not increase during the VR task in the present sample, *t*(64) = 0.75, *p* = 0.454, *d* = 0.13.

### 3.2 Control Tasks

#### 3.2.1 Web‐Based Stop‐Signal Reaction Time and Chocolate Intake

The stop‐signal task had to be repeated by *n* = 5 participants to achieve an adequate stopping probability between 25%–75%. Furthermore, data from the web‐based task could not be uniquely mapped for *n* = 5 participants due to ambiguous codes. For two participants, the RT on unsuccessful stop trials was numerically longer than RT on go trials and thus we did not estimate SSRT (cf. Verbruggen et al. 2019).

The SSRT in chocolate trials was not significantly different from SSRT in neutral trials, *t*(66) = 0.98, *p* = 0.329, *d* = 0.12 (see Table 3). There was no significant association between chocolate intake and SSRT in chocolate trials, *r*(66) = −0.21, *p* = 0.093, nor in neutral trials, *r*(66) = −0.13, *p* = 0.302. Reaction time, false alarms and stop‐signal delay are reported in Table 3.

**TABLE 3:** Results from the web‐based stop‐signal task (four top rows), the virtual reality task (bottom rows) and raw associations with chocolate intake (chocolate trials and shoe trials).

	Trial‐type	Condition *M* (SD)				
Parameter		Chocolate	Reference	*t/F*	*r*_CHOC_	*r*_REF_
Stop‐signal reaction time [ms]		326 (68)	320 (73)	0.98	−0.21	−0.13
Reaction time [ms]		693 (154)	659 (151)	7.94***	0.10	0.06
False alarms/stop errors [%]		46 (5)	47 (6)	1.97	0.15	0.05
Stop‐signal delay		378 (154)	342 (144)	5.13***	0.22	0.14
False alarms/stop errors [%]	Stop	24 (14)	29 (14)	4.48*	0.06	0.08
Spatial displacement (approach) [cm]	Stop	54.6 (3.1)	56.6 (3.2)	0.18	−0.01	0.04
Peak velocity [m/s]	Go	2.09 (0.31)	2.11 (0.31)	17.10***	−0.18	−0.14
Peak acceleration [m/s²]	Go	2.02 (0.30)	2.05 (0.30)	14.84***	−0.17	−0.14
Time to stop [ms]	Stop	247 (72)	260 (64)	6.24*	0.07	0.04
Initial gaze [ms]	Go	396 (142)	289 (105)	142.8***	0.12	0.18
Total gaze [ms]	Go	677 (211)	461 (174)	233.2***	0.19	0.25

*Note:* For each parameter, the mean (SD) value of chocolate and reference trial is reported. The difference between the two conditions was tested by dependent‐sample *t*‐tests. Finally, raw correlations with chocolate intake are reported separately for chocolate and reference trials.

*p < 0.05, **p < 0.01, ***p < 0.001.

#### 3.2.2 Web‐Based Implicit Chocolate Approach and Chocolate Intake

In the single‐category IAT, the same *n* = 5 participants' data could not be mapped to chocolate intake as in the web‐based stop‐signal task. Based on their overall accuracy, the algorithm for computation of D‐score furthermore rejected data from *n* = 4 participants. Thus, D‐score could be calculated for all remaining participants.

Overall, a negative D‐score was measured by the task (*M* = −0.137, SD = 0.22), indicating faster responses in the incompatible test block (i.e., avoidance words paired with chocolate pictures) compared to the compatible test block. The negative IAT effect reflected overall slightly faster responses if chocolate was paired with avoidance (*M* = 603 ms, SD = 244) compared to approach words (*M* = 609 ms, SD = 220 ms). In the present sample, the negative IAT effect was significantly different from zero, *t*(64) = −5.11, *p* < 0.001, *d* = −0.63. There was no significant association between chocolate intake and chocolate‐related implicit approach‐avoidance classifications, *r*(63) = 0.09, *p* = 0.456.

### 3.3 Biometric Parameters in the VR Stopping Task

Biometric data of all *n* = 74 participants was available and included between 51.9%–100% successful stop‐trials, yielding 25–50 correct stop‐trials per category and participant for trajectory analyses. Participants committed lower stop error rates in chocolate trials (*M* = 24%, SD = 14%) relative to neutral trials (*M* = 29%, SD = 14%), *t*(73) = 6.49, *p* < 0.001, *d* = 0.75.

**FIGURE 2:**
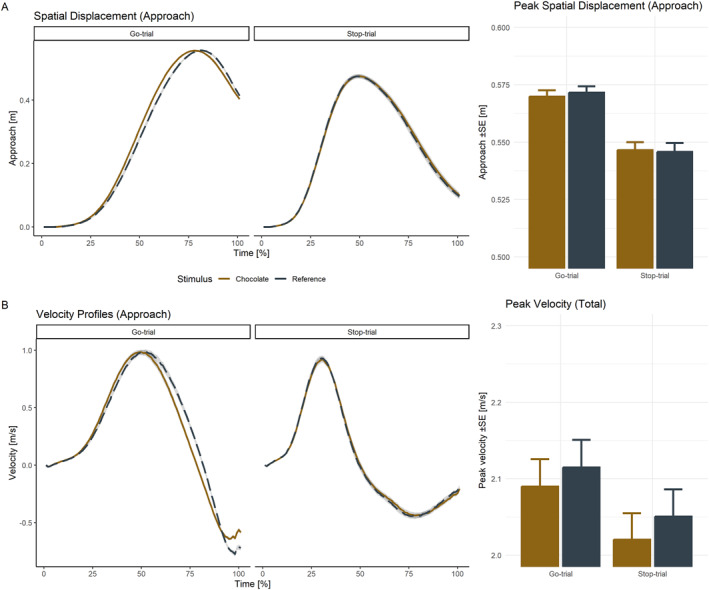
Group‐level biometric trajectories in go‐trials and stop‐trials to chocolate stimuli (brown straight line) and reference objects (shoes; dark‐blue dotted line). Panel A shows standardized continuous (left side) and peak spatial displacement (right side). Panel B shows velocity profiles in approach (left side) and total peak velocity (right side).

We next analysed spatial displacement in the approach dimension with the fixed effects Category (chocolate vs. neutral) and Trial‐type (go‐trial vs. stop‐trial). Correct go‐ and stop‐trials differed significantly in terms of maximum spatial displacement in movement approach, *F*(1, 73) = 114.71, *p* < 0.001*, n*_*p*_^2^ = 0.61. The movement profiles derived from the kinematic trajectories showed the expected approach towards chocolate and control objects (Figure 2) and a relatively late interruption of movements at a mean distance of 54.6 cm (SD = 3.1 cm). On average, movements were thus terminated at only 2.45 cm before the virtual object. Chocolate and control objects were stopped equally effectively: Peak spatial displacement showed neither a main effect of Category, *F*(1, 73) = 0.18*, p* = 0.671*, n*_*p*_^2^ < 0.01, nor a two‐way interaction of Category × Trial‐type*, F*(1, 73) = 1.51, *p* = 0.223, *n*_*p*_^2^ = 0.02 (see Figure 2A).

Movement derivatives (peak velocity, peak acceleration, peak acceleration times) also caught the effects of stopping in the main effect of Trial‐type, *F*s ≥ 55.0, *p*s < 0.001, see Table 3. Furthermore, a few stimulus effects of Category emerged across both stop‐ and go‐trials: Statistically, kinematic variables showed a less rapid movement towards chocolate, as evidenced by a main effect of Category in peak acceleration, *F*(1, 73) = 14.84, *p* < 0.001, *n*_*p*_^2^ = 0.17 and in peak velocity, *F*(1, 73) = 17.10, *p* < 0.001, *n*_*p*_^2^ = 0.19 (see Figure 2B). The stimulus effect in kinematic variables was not qualified by a two‐way interaction with Trial‐type, *F*s < 0.22, *p*s > 0.639.

In the temporal domain, moreover, the directly measured stopping latency (i.e., *Time to Stop*) was shorter for chocolate (*M* = 247 ms, SD = 71 ms) than for control objects (*M* = 260 ms, SD = 64 ms), *F*(1, 73) = 6.24, *p* = 0.015, *n*_*p*_^2^ = 0.08 (see Figure 2B, right panel).

To summarise, the biometric parameters at the group level show a more controlled and delayed movement towards chocolate, evidenced by lower peak acceleration and peak velocity, but faster stopping latency. Lower stop error rates also indicate an increased inhibitory control to chocolate compared to the reference stimuli, similar to the results obtained in the web‐based stop‐signal task.

Regarding the predictive validity of biometric parameters for chocolate intake, we did not observe statistically significant correlations with chocolate intake, see Table 3.

### 3.4 Eye‐Tracking

Relative to shoes, eye‐tracking measures of attention showed longer initial dwell time to chocolate, *F*(1, 73) = 142.76, *p* < 0.001, *n*_*p*_^2^ = 0.66 and longer total dwell time to chocolate, *F*(1, 73) = 233.20, *p* < 0.001, *n*_*p*_^2^ = 0.76 (see Table 2). As expected by the task design, total fixation duration was longer during stop‐trials, *F*(1, 73) = 163.43, *p* < 0.001, *n*_*p*_^2^ = 0.69, but the initial fixation duration did not differ between go‐ and stop‐trials, *F*(1, 73) = 1.26, *p* = 0.265, *n*_*p*_^2^ = 0.02. There were no significant two‐way interactions between the factors Category and Trial‐Type, *F*s < 3.54, *p*s > 0.064. Regarding their predictive validity, we did not observe statistically significant correlations with chocolate intake, see Table 3.

### 3.5 Feature Selection in the Elastic Net Regression (Exploratory)

**FIGURE 3:**
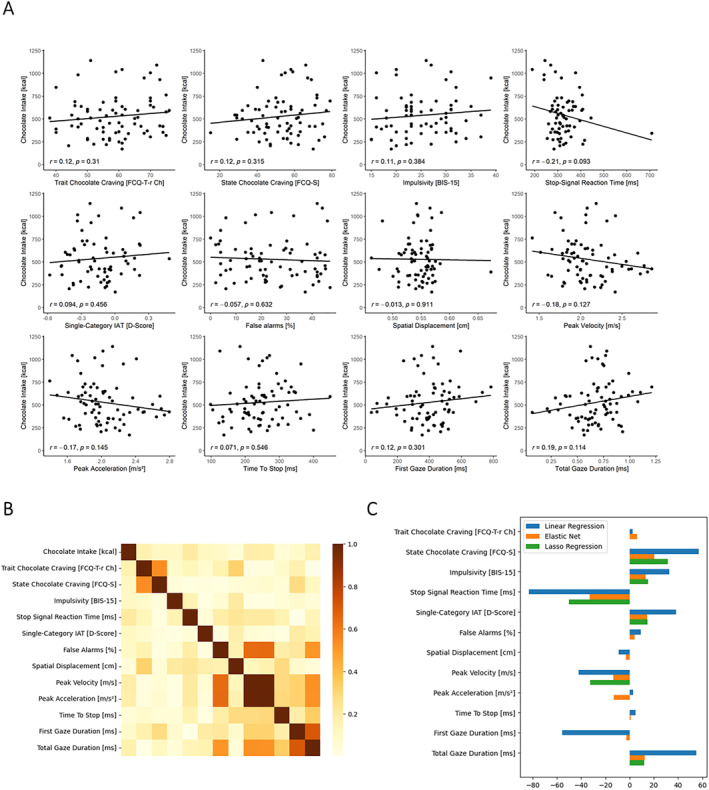
Elastic net regression. (A) Raw correlations with chocolate intake, (B) inter‐correlations of the predictors and (C) standardized weights in linear, elastic net and Lasso regression (blue, orange and green bars, respectively).

We performed further exploratory, data‐driven analyses for feature selection, that is, elastic net regression and Lasso regression (see supplement for details). The aim of the analysis was to determine unique predictors of chocolate intake, by submitting all subjective, behavioural and biometric predictors concurrently to the elastic net. Because the elastic net did not zero out any of the weights despite their high intercorrelations (see Figure 3), Lasso regression was additionally employed, leading to a sparser model. However, the model fit was still similar to both the linear regression and the elastic net model with balanced feature selection (in‐sample MAE: 165.83 kcal; out‐of‐sample MAE: 184.65 kcal). The resulting model selected trait chocolate craving, trait impulsivity, SSRT, implicit avoidance, peak velocity and total dwell time, as concurrent predictors of chocolate intake.

## 4 Discussion

This study tested the ability of biometric parameters of response stopping to predict subsequent chocolate food intake. In line with the accumulating literature on direct measures of inhibitory control (Leontyev and Yamauchi 2019; Schroeder et al. 2023, 2024) our VR task clearly separated stop‐ from go‐trials and revealed several subtle behavioural effects of chocolate stimuli at the group level.

Against our expectations, the strongest predictors of chocolate intake were the indirectly measured SSRT and stop‐signal delay in a web‐based control task, performed on a separate day under uncontrolled conditions, and the total gaze duration to non‐chocolate reference objects. A machine learning analysis (i.e., Lasso regression) further selected peak velocity, implicit chocolate approach and chocolate craving as distinct predictors of chocolate intake, with a very rigorous penalty. However, even for these associations small effect sizes were found. In this regard, our findings align with low correlations between spontaneous food intake/food choice and inhibitory control (Bartholdy et al. 2016; McGreen et al. 2023). At the same time, the direction of the correlation is not consistent. All but one previous study in preschool children (Levitan et al. 2015), which was subsumed in the meta‐analysis (McGreen et al. 2023), reported higher food intake with lower inhibitory control, as indicated by *higher* SSRT. Descriptively, our present findings indicated higher chocolate intake with better inhibitory control, as indicated by *lower* SSRT.

Thus, SSRT particularly including chocolate‐trials was inversely associated with chocolate intake in the present study.^2^ Surprisingly, chocolate consumption was most pronounced in those individuals with the most outstanding inhibitory control abilities. Although there were other previous studies that reported non‐significant associations between food consumption and inhibitory control (Guerrieri, Nederkoorn, and Jansen 2007; Haynes et al. 2015; Hermans et al. 2013), previous findings rather indicated higher consumption with lower inhibitory control (Nederkoorn et al. 2009) even in non‐clinical samples (Guerrieri, Nederkoorn, Stankiewicz, et al. 2007; Nederkoorn et al. 2009). We measured inhibitory control specifically to chocolate, following the concept of food‐specific inhibition as outlined in previous work (Bartholdy et al. 2016). In the present data, however, neither the parameters in chocolate trials nor in shoe trials were substantially predictive of chocolate intake and both suggested the inverse relationship.

Of course, we can only speculate about the underlying reasons for this surprizing finding. Certainly, the recruitment of (mild‐to‐modest) trait chocolate cravers for a week of chocolate abstinence may have induced a specific state of chocolate craving in the present study. Noteworthy, accordingly, consumption during the chocolate‐modified bogus taste test was relatively high (i.e., up to 1141 kcal). Moreover, chocolate can be considered a hedonic food (Blechert et al. 2014; Lowe and Butryn 2007), and thereby possibly encounters more inhibition in every‐day life, compared to other food. At the same time, for crisps, a strong association in the expected direction of higher food intake with lower inhibitory control was reported (Houben and Jansen 2014). More research is needed to uncover the underlying discrepancies between inhibitory control for hedonic (i.e., chocolate and crisps), high‐calorie savoury and low‐calorie food and of the successful versus unsuccessful abstinence from high‐calorie food in clinical and more extreme risk cravers.

### 4.1 Late‐Stage and Early‐Stage Inhibitory Control

We designed and presented the VR task as a measure of late‐stage inhibitory control since it offers the unique advantage of recording prolonged and interrupted motor responses. Thus, the results derived from the task are a direct quantitative measure of action cancelation, with objective evidence that the motor response had been initiated (e.g., in the dynamic starting line and the continuous hand position recordings).

Notwithstanding the focus on late‐stage inhibition in the present work, it is important to also consider early‐stage processes in food‐related inhibitory control. For example, there is ample evidence of the interaction between food and interference control in Flanker and Stroop tasks (Smith et al. 2018). In obesity and overweight, inhibition deficits have been described in a large variety of (food‐related) executive function tasks (Yang et al. 2018). However, fundamental research has recently outlined mechanistic differences to be detected already within the classical response inhibition paradigms (i.e., go‐/no‐go and stop signal tasks; Raud et al. 2020). Furthermore, the VR version and a matched two‐dimensional stop‐signal task were differentially related to chocolate craving and depression, respectively (Schroeder et al. 2023). Future research is needed to better understand the cognitive processes underlying different inhibition paradigms and their relations with food craving and intake.

### 4.2 Limitations

We initially anticipated a larger effect size in our preregistration and allocation of resources than was recently outlined in a meta‐analysis across go/no‐go and stop‐signal tasks with food intake and food choice (McGreen et al. 2023). Thus, although a reasonable sample of trait chocolate cravers was recruited, the statistical power to detect small correlations was limited. Moreover, although the elastic net is capable of modelling a high number of predictors, the quality of the estimation is suboptimal. Surprisingly, the largest (negative) association with chocolate intake was observed in the web‐based control task for inhibitory control, which was much less standardized than the VR task in terms of chocolate abstinence, timing, setting, satiety. It is possible that participants self‐selected their optimal time‐point for a web‐based task on chocolate, although state chocolate craving before the web‐based task was moderate and very variable.

Regarding the recruited sample, different results could be expected for clinical populations or extreme groups of chocolate craving. It is important to emphasise that all participants self‐selected for a chocolate fasting period and had a BMI below 30 kg/m^2^. In this population, chocolate craving and consumption may not constitute a serious issue related to overeating, but a hedonic experience acted on one's inhibitory control abilities. Accordingly, it is possible that individuals with overweight, binge eating or loss of control over eating might encounter more inhibitory control difficulties with chocolate. A further consideration pertains to the selection of non‐obese individuals capable of abstaining from chocolate consumption for a week. We could assume that participants capable of this requirement were individuals who, in their daily lives, are able to exercise inhibitory control over chocolate. It follows that, in the unique experimental setting created by the bogus taste test and the previous abstinence period, it is possible that individuals with high craving behaved differently from their everyday life. Thus, having better inhibitory control in everyday settings might have, paradoxically, led to more chocolate intake in the experiment. Finally, age of participants was also limited due to its correlation with inhibitory control (Schroeder et al. 2021); however, it is not clear if the results generalise to representative samples of chocolate cravers and food cravers across the life span.

An ongoing debate revolves around hyperparameter optimization in elastic net. Often, it is decided a priori to use fixed parameters and avoid overfitting. In the present case, we chose a more data‐driven approach and optimised the α–hyperparameter via grid search. In many situations this approach can lead to model overfit. The fact that both in‐sample as well as out‐of‐sample errors were still high point to the existence of additional variables that we did not consider. Moreover, the error rates (both types of errors) qualify additional concerns about overfitting with the present data. A final important technical consideration is that predictor shrinkage was low and only Lasso regression performed feature selection. Since Lasso regression does not consider groups of predictors, in particular the interpretation of the choice of variables selected among the kinematic parameters remains slightly speculative and the selection of peak velocity might be driven simply by its large individual coefficient weight, relative to the other biometric predictor variables.

## 5 Conclusion

We provide further evidence for enhanced chocolate‐specific inhibitory control that enables a controlled handling and consumption of craved food. In measures derived from VR, shorter stopping times and higher stopping rates indicate superior stopping abilities to chocolate stimuli compared to a reference category of 3D objects, that is, shoes. Regarding chocolate intake, a small negative correlation suggested more consumption in chocolate cravers with superior inhibitory control abilities, contrary to our hypotheses. The findings may indicate a fundamental cognitive mechanism that enables hedonic chocolate encounters.

## Ethics Statement

The authors assert that all procedures contributing to this work comply with the ethical standards of the relevant national and institutional committees on human experimentation and with the Helsinki Declaration of 1975, as revised in 2008.

## Conflicts of Interest

The authors declare no conflicts of interest.

## Supporting information

Supporting Information S1

## Data Availability

The data that support the findings of this study are openly available in Open Science Framework at https://osf.io/m3yvt/, reference number m3yvt.

## Endnotes

^1^
Only one previous study reported a small negative association between snack intake and inhibitory control in 48‐month old children (Levitan et al. 2015)

^2^
A further sensitivity analysis substantiated this pattern and even if extreme outliers with SSRT > 600 ms were removed from the data (see Figure 3), the correlation with chocolate intake was hardly reduced from *r* = −0.21 in the full data set to *r* = −0.19 in the sensitivity analysis.
