# Supplementary material for: Noshing on Chocolate, I Can Do That: Increased Chocolate Consumption in the Chocolate‐Modified Bogus Taste Test With Better and Not Worse Inhibitory Control
Source: Eur Eat Disord Rev. 2025 May 19;33(5):1085–98. doi: 10.1002/erv.3206 (PMC12319143; doi:10.1002/erv.3206)
Supplement: Supplementary file 1 — Supporting Information S1 [file ERV-33-1085-s001.docx]

**Supplementary materials for *Noshing on chocolate, I can do that: Increased chocolate consumption in the chocolate-modified bogus taste test with better and not worse inhibitory control***

Supplementary materials

[Supplementary methods 1](#_Toc187682355)

[Power analyses and sample size justification 1](#_Toc187682356)

[Virtual reality task 1](#_Toc187682357)

[VR Stimuli 2](#_Toc187682358)

[Control tasks 2](#_Toc187682359)

[Web-based stop-signal task 3](#_Toc187682360)

[Web-based single-category implicit association test of chocolate approach 5](#_Toc187682361)

[Data preprocessing of web-based control tasks 6](#_Toc187682362)

[Questionnaires 7](#_Toc187682363)

## Supplementary methods

### Power analyses and sample size justification

The sample size was justified by available resource during the second and thrird waves of the Corona pandemic and by two a priori power analyses, indicating possible scenarios for data collection in the chocolate intake test. The lower-end power analysis (see pre-registration) indicated a minimum sample of N=67 to investigate medium-sized positive correlations (ρ = 0.3) between behavioral parameters and chocolate intake variability in the bogus taste test with acceptable statistical power (1 – β = 0.8) and at α = 0.05.

### Virtual reality task

The handheld controller was implemented as a white right hand in the virtual environment and the position and movement of the controller continuously updated at refresh rate (120 Hz). The hand also provided color-based trial feedback after correct (green) and incorrect (red) responses.

Throughout the VR task, participants were seated in a chair with enough space to move their arms and hands freely around them. Two opposing lighthouse boxes were firmly installed in the room to capture the movement data. The experiment was implemented using Unity3D 2018.4.17f1, the SteamVR 1.2 library, and custom C# code.

In brief, the task resembled a conventional stop-signal task and consisted of 300 go-trials and 100 stop-trials, with a salient stop-signal directly embedded in the virtual environment. Participants could start a trial by putting their right hand into a starting position (a schematic hand shown at the anterior edge of a virtual table). Once they fixated a red sphere placed in the target position at approximately 53cm distance and 20cm above the starting position (confirmed by the integrated eye-tracking), the target stimulus (food or shoe object) replaced the fixation object and participants could start moving towards the target. Fixation and target objects were placed above a virtual silver tray as part of the environment. Participants were tasked to pick up the target with their right hand and place it onto one of two plates (at the left/right sides of the starting position), dependent on whether the target object depicted chocolate or a shoe. However, in 25% of the trials, a stop-signal was shown after movement onset (that is, after participants had started their movement and crossed an imaginary line beyond the starting position; dynamic starting line (Scherbaum & Kieslich, 2018)). The stop-signal was a color change in of the silver tray, turning it into blue. The stop-signal delay was adjusted individually for the chocolate and neutral target category after every stop-trial in steps of 50 ms (minimum: 50 ms; maximum: 700 ms).

The virtual environment was a rebuilt snacking test room, in order to maximize resemblance to the later context of food intake. The virtual and real laboratory were closely matched (see Figure 1, main text).

### VR Stimuli

We used photorealistic 3D models of chocolate as target stimuli. The chocolate stimuli depicted the same objects that were later served in the BTT. Most of the chocolates were also used in a previous study (Schroeder, Mayer, et al., 2023), except for chocolate cookies and chocolate lentils . As control category, we selected shoes (e.g., sports shoe, ballerina shoe, high heels) as a clear comparison category with comparable attractiveness , and with potentially comparable hedonic value (Werthmann et al., 2014). Moreover, previous research showed larger effects for explicit food classification in an approach-avoidance task (Lender et al., 2018). All stimuli were selected and slightly adjusted in external 3D software as closely as possible regarding their total volume, color, texture, and affordance.

### Control tasks

We collected data from two web-based control tasks, reflecting more conventional behavioral parameters. These tasks were implemented using jsPsych (de Leeuw, 2015). Participants received an invitation for self-paced recording of the tasks within 48 hours before their lab appointment (mean = 26.3 (SD = 18.4) hours before the BTT^^[[1]](#footnote-1)^^).

#### Web-based stop-signal task

The web-based stop-signal task included 400 trials (100 stop-trials) with pictures of the same chocolate and shoe cues as used in the VR task. We implemented the task following recent consensus recommendations (Verbruggen et al., 2019) and close to our previous chocolate-specific implementation (Schroeder, Mayer, et al., 2023). In brief, the stop-signal task required participants to determine the position of a picture in one of two windows by responding with their left or right index finger. In 25% of the trials, an additional stop-signal appeared on the screen (a blue frame surrounding the target picture) after a variable delay (i.e., stop-signal delay). In stop-trials, participants are instructed to withhold their response.

The present web-based stop-signal task was designed as closely as possible to the VR task and included a chocolate-relevant go-decision (left or right key press for chocolate vs. shoe stimuli) and an independent stop-signal delay tracking for chocolate and shoe cues. That is, after every correct or incorrect stop-signal trial, the stop-signal delay for the displayed stimulus category was decreased or increased by 50 ms (with a minimum stop-signal delay of 50 ms and a maximum of 700 ms). We used two-dimensional screenshots of the same chocolate and shoe stimuli as displayed in the VR task, and the same chocolate stimuli as later consumed during the BTT (see Figure 1, main text, and Supplementary Figure 1).

The dependent variable from the stop-signal task was the estimation of stop-signal reaction time as an established measure of inhibitory control (Schroeder, Farshad, et al., 2023; Verbruggen et al., 2019). Stop signal reaction time indicated the time required to stop an activated response and is calculated from the difference between the response time in go-trials and the mean stop-signal delay. We used the integration method to determine stop-signal reaction time with replacement of response omissions at the upper end of the distribution of go-trial response times (Verbruggen et al., 2019). To avoid the use of a waiting strategy to perform correct on all stop-trials, all participants were informed about the requirement to achieve a stopping probability of approximately 50% in the task. In line with the recommendation from the SST consensus, we did not estimate stop signal reaction time for stopping probabilities lower than 25% or higher than 75%; participants with exceedingly high stopping probabilities were asked to repeat the task (5 recordings).

***Supplementary Figure 1.***

*Example go- and stop-trials of the web-based stop-signal task (top) and the VR task (bottom).*

*
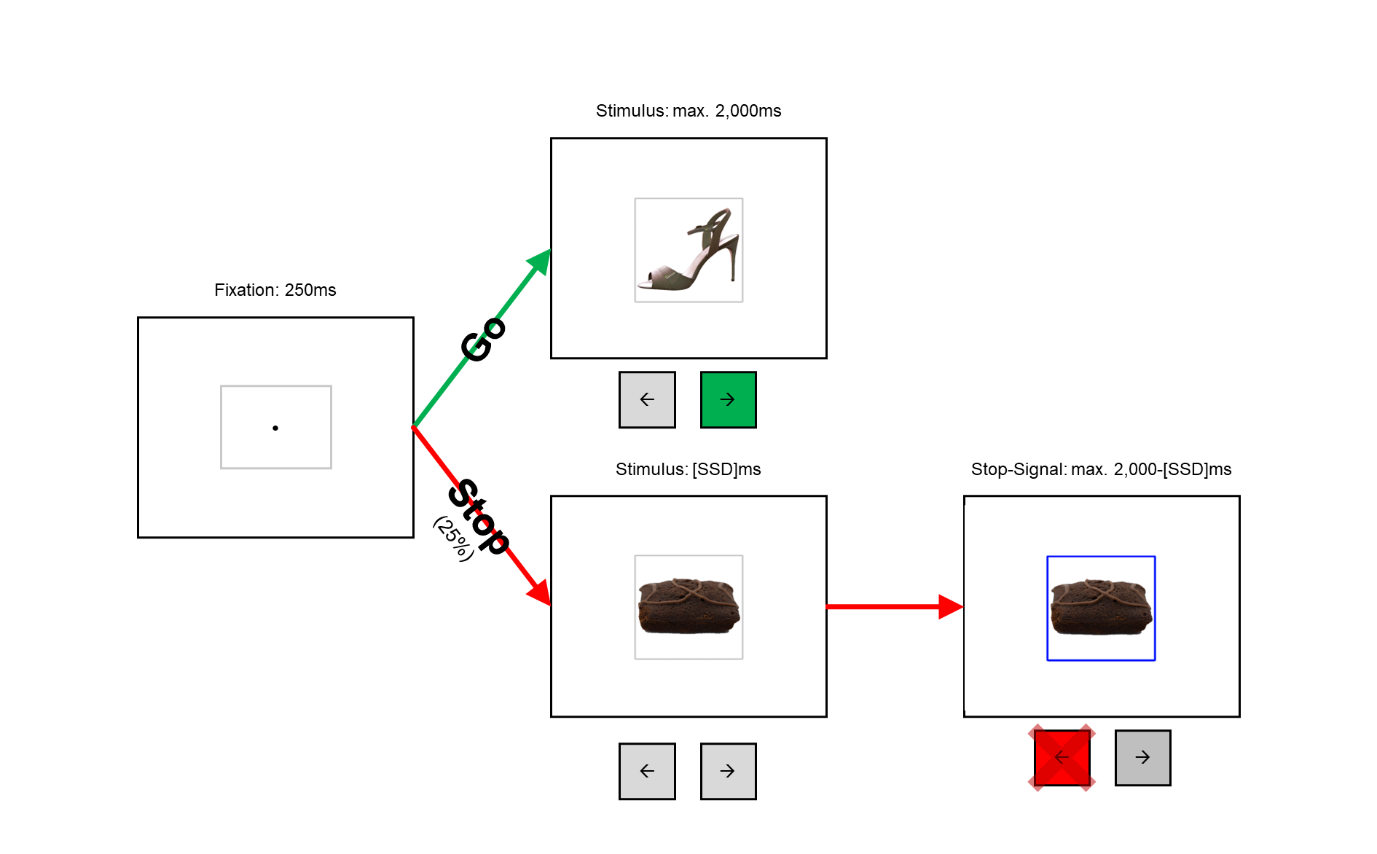
*


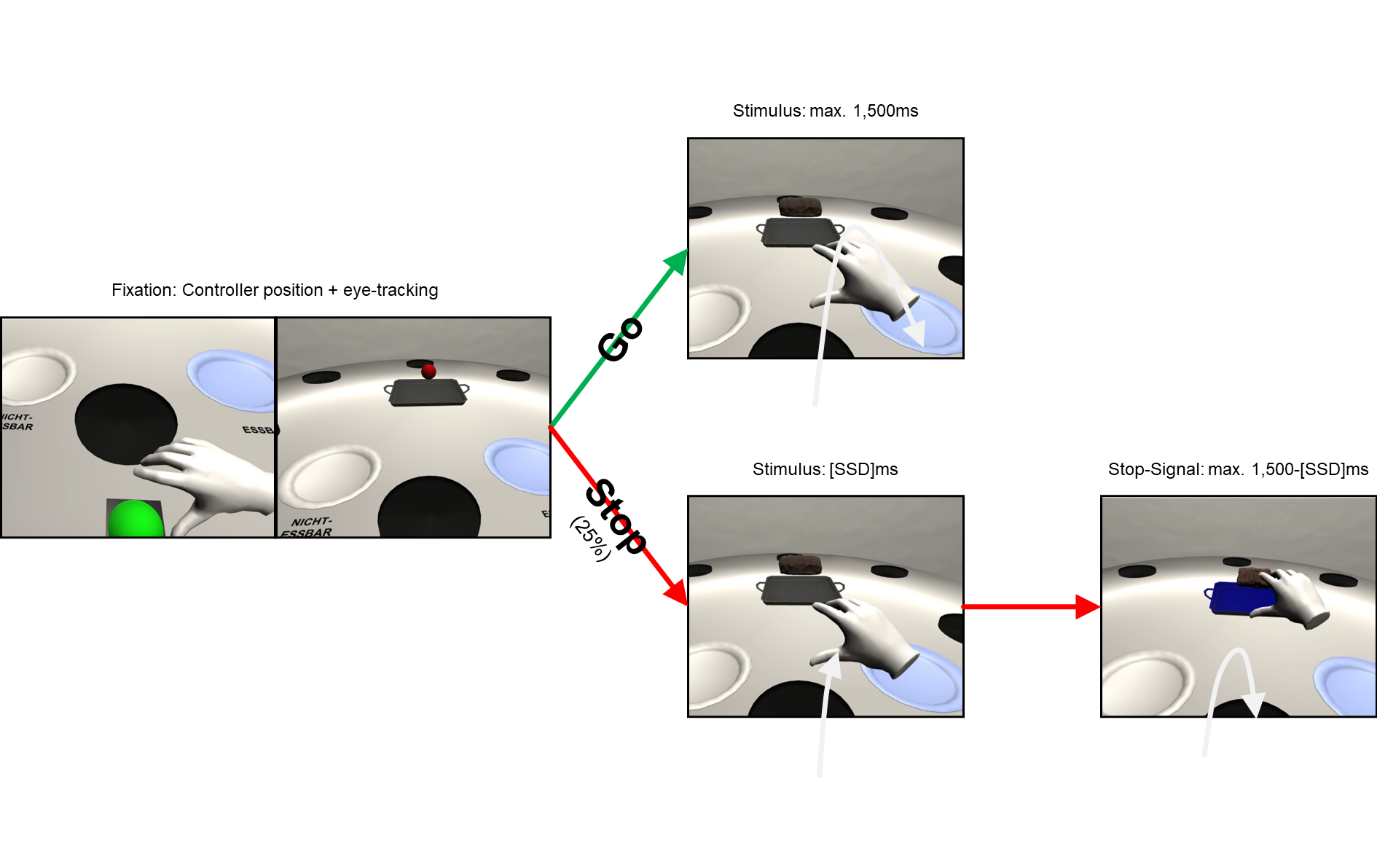


#### Web-based single-category implicit association test of chocolate approach

To obtain a second control measure that was not specifically linked to inhibitory control, participants answered a single-category implicit association test with the same chocolate pictures on implicit chocolate-related approach-avoidance classifications (Karpinski & Steinman, 2006; Richetin et al., 2007; but see Klauer et al., 2010). Originally, the single-category implicit association test was conceived to measure the strength of evaluative associations at the implicit domain, that is, that are not directly reported or observed. The task subsequently introduces the target and evaluation dimensions. After practice, both dimensions are evaluated by using the same key press in all possible combinations, in separate test blocks (e.g., chocolate and approach vs. shoe and approach). The difference in reaction time between blocks is analyzed. The core idea of the task is, if a relative implicit association exists between target and the classification dimension (e.g., between chocolate and approach), reaction time should be faster for the test condition that maps the compatible polarities compared to the test condition that maps the incompatible polarities on the same response key. The recommended dependent variable from the task is the D-score, which takes into account the standardized differences between the two test blocks and gives instructions on the detection of outlier values D-score (see Karpinski & Steinmann, 2006; Greenwald, Nosek, & Banaji, 2003). Essentially, the difference of RT between compatible and incompatible test blocks relative to their standard deviation reflects the D-score.

Example trials of the task are shown in Supplementary Figure 2. In the present implementation, participants were instructed to press the ‘E’ or ‘P’ key with their left-hand or right-hand index finger, respectively, in response to the central picture or word stimuli. The attribute labels were introduced first and the categories “to approach” and “to avoid” were continuously displayed on the top left and right side of the screen (e.g., to take or to remove). Next, chocolate pictures were introduced as the target category and participants were randomly confronted with stimuli (approach: “greifen/grasp”, ”nehmen/take”,”wollen/want”,”wünschen/wish”; avoid: “meiden/avoid”,”lassen/leave”, “wegschieben/push”, “entfernen/remove”, or pictures of chocolate). In the first block, approach words and chocolate were both classified with the left-hand key, and avoidance words were classified with the right-hand key. The assignment of chocolate was changed to the right-hand key (i.e., together with words of avoidance) in the reverse practice block and the subsequent incompatible test block. Given that the sequence of compatible and incompatible blocks can influence the magnitude of the IAT effect (e.g., Schroeder et al., 2018), we did not vary the sequence in order to maximize individual differences relative to chocolate-approach related cognitive processing as captured by the task. The order of stimuli within the blocks was randomized and balanced. Both test blocks comprised 72 trials in random order. The trials had a response deadline of 1,500 ms and feedback for correct, incorrect or slow responses was briefly displayed for 150 ms after every trial. There were 18 additional practice trials for familiarization (i.e., without chocolate pictures), 24 additional practice trials before the compatible test and 24 reverse practice trials before the incompatible test block.

***Supplementary Figure 2.***

*Example trials of a compatible (left) and incompatible test block (right) in the single-category implicit association test.*


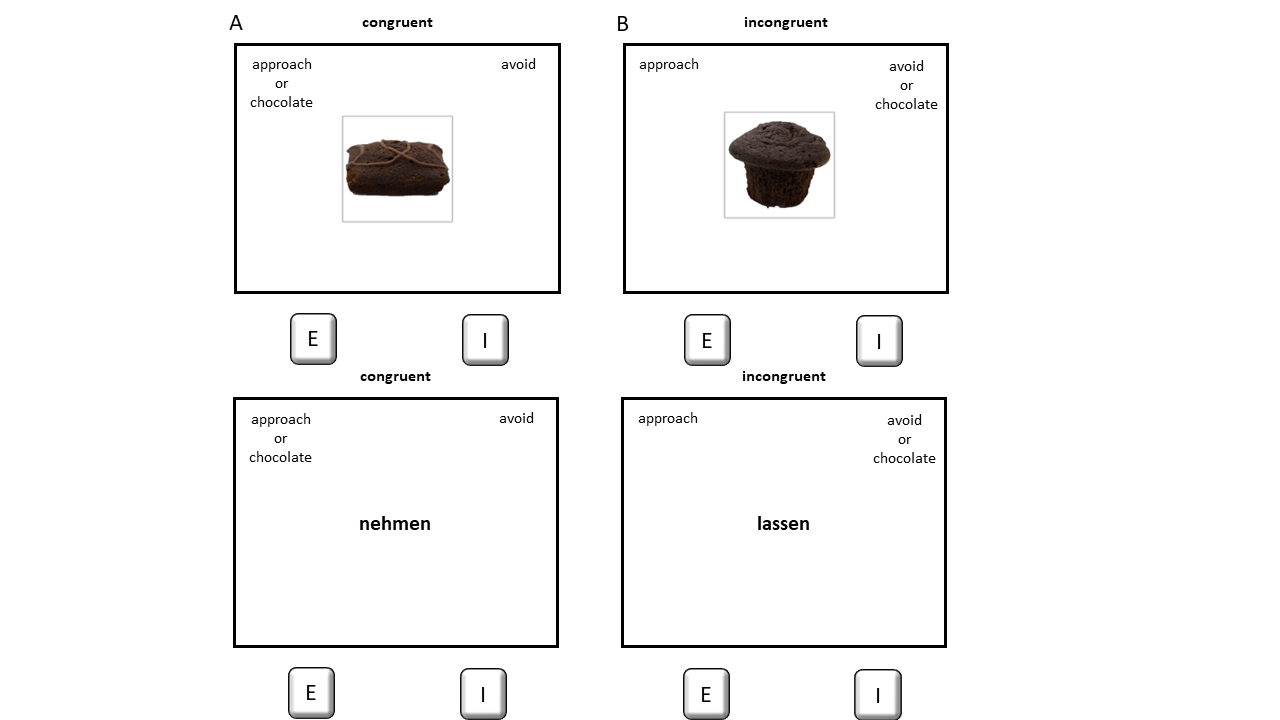


**^Note. Nehmen = take; lassen = leave^**

#### Data preprocessing of web-based control tasks

We administered standardized scoring algorithms for extraction of outcome variables from the web-based tasks. For the stop-signal task, we used the integration algorithm with replacement of response omissions to compute the stop-signal reaction time (Verbruggen et al., 2019). Given the indirect estimation of stopping latency in the stop-signal reaction time, the consensus is to include only participants with a response probability in stop trials between 25-75%. Furthermore, we checked if RTs on failed stop-trials were longer than RTs on go-trials, which is considered a second criterion for the validity of the horse race model underlying the SSRT estimation. The stop-signal reaction time was computed as:

$$SSRT = RTn - M(SSD)$$

with *n* = *p*(response|stop) * number of trials, and RT = distribution of all RTs in go trials, and *M*(SSD) as the mean SSD across all relevant stop-trials (i.e., across chocolate- or shoe-stop trials).

From the single-category IAT, we computed the D-score (see Karpinski & Steinmann, 2006; Greenwald, Nosek, & Banaji, 2003), as implemented in the *implicitMeasures* package for R (Epifania et al., 2020). Essentially, the difference of RT in compatible and incompatible test blocks relative to their standard deviation reflects the D-score. The *compute_sciat()* function implements scoring functions of the algorithm, i.e., rejection of participants with more than 25% errors, and the deletion of fast (<350 ms) and slow (>10000 ms) trials.

## Questionnaires

*FCQ-T-r-Ch.* The Chocolate version of the Food Cravings Questionnaire (reduced; trait version) was administered to assess trait chocolate craving during the online screening and determine the chocolate-craving sample (Meule & Hormes, 2015). This version included 15 items of chocolate craving with a two-factor structure to differentiate hunger for chocolate and craving. Internal consistency in this sample was acceptable (Cronbach’s α = .89).

*FCQ-S-r-Ch.* The State variant of the Chocolate version of the Food Cravings Questionnaire (reduced) was admitted before and after virtual reality to assess state chocolate craving (Meule & Hormes, 2015). The questionnaire included 15 items of chocolate craving in the present moment and showed excellent internal consistency (Cronbach’s α = .91 (pre) and α = .93 (post)).

*VAS.* A single-item visual analogue scale on chocolate craving was answered before the web-based control tasks (range: 0-100).

*EDE-Q.* The Eating Disorder Examination-Questionnaire (EDE-Q) is a 28-item self-report measure of eating disorder symptoms (Hilbert et al., 2007). The EDE-Q has excellent psychometric properties, normative data for populations are available (Quick & Byrd-Bredbenner, 2013). Internal consistency of the total EDE-Q score in this sample was excellent (Cronbach’s α = .91).

*PHQ-9.* We used the 9-item depression module of the Patient-Health-Questionnaire (PHQ-9) (Gräfe et al., 2004) to screen for depressive symptomatology with a good subthreshold sensitivity. The brief PHQ-9 has shown good psychometric properties and sensitivity in healthy student populations (Zhou et al., 2020). Internal consistency in this sample was not good (Cronbach’s α = .58).

*DEBQ.* The Dutch Eating Behavior Questionnaire is a self-report assessment for eating styles. Subscales include eating restrained eating, emotional eating, and external eating (van Strien et al., 1986). Internal consistency in this sample was acceptable (Cronbach’s α > .76).

*Barrat Impulsivity Scales 15.* The Barratt Impulsiveness Scale (BIS-15) is a validated 15-item short-version of trait impulsivity (Meule et al., 2011). Questionnaire items assess impulsiveness in several everyday situations (e.g., “I act spontaneously”, “I plan for the future [inverted]”). Internal consistency of the global score was acceptable in this sample (Cronbach’s α = .78).

*Handedness.* Right-handedness was confirmed by the 10-item Edinburgh Handedness Inventory (EHI). Items ask for preferential hand use in typical everyday scenarios. The resulting laterality quotient (LQ) was computed (range: -100 (strong left-handedness) – +100 (strong right-handedness)) and classified all participants as right-handed (minimum LQ = +57.9, mean LQ = 96.4).

# Supplementary results

## Elastic net regression

Many of the predictors from biometric recordings were intercorrelated and it was unclear if any of the measures yield predictive validity, given the overall low raw correlations with chocolate intake. As conventional linear regression models suffer from overfitting due to inflated weights when predictors are correlated (Kalnins, 2022), we extended our preregistered analyses with a regularized regression model (elastic net, Zou & Hastie, 2005). The elastic net penalizes large weights by adding the L1 and L2 norm of the weight vector to the cost function of the regression. While this consciously introduced bias can lead to a slightly negative impact on the fit of the model on the training data (but not the test data), the weight shrinkage alleviates overfitting when predictors are correlated. Additionally, the elastic net also allows for feature selection by potentially zeroing some coefficients. It contains two hyperparameters: α which determines the total amount of regularization and the *ratio_L1_* parameter which determines the ratio of L1 vs. L2 penalty. A higher *ratio_L1_* leads to more rigorous feature selection.

To choose the best hyperparameters, a systematic grid search was performed (Hutter et al., 2019). For α, a grid comprised of logarithmic steps from 0 to 1 and linear steps from 2 to 20 with a stepsize of 2 was used as part of an automated Leave-One-Out Cross Validation (LOO-CV) procedure (Celisse, 2014). LOO-CV trains the model on *n*_train_−1 samples, using the remaining sample to assess the out-of sample performance. Each sample is left out once, and the error is averaged over all of these splits. Through this, the training set can almost be left intact during the validation procedure, leading to less bias when estimating the out-of-sample error especially in scenarios with only limited amounts of data (Zhang & Yang, 2015). Due to its robustness to outliers, performance was assessed via the mean absolute error (MAE) both within-sample and via LOO-CV for estimating the performance on unseen data. In order to contrast the performance of the elastic net, a unregularized but otherwise identical linear regression model was also fit to the data, which is expected to yield better in-sample MAE but worse out-of-sample generalization.

We examined the prediction of chocolate intake from all relevant parameters, including the SSRT as gathered in the web-based stop-signal task, and the chocolate-approach association as gathered in the implicit association task. Moreover, we concurrently submitted all preregistered biometric parameters from chocolate trials in virtual reality, as well as eye-tracking parameters, subjective chocolate craving, and trait impulsivity to the regression models. To promote more severe feature selection a relatively high *ratio_L1_* was set to 0.8. As expected, the conventional linear regression performed marginally better than the elastic net in-sample (MAE of 164.34 kcal vs MAE of 168.27 kcal), but exhibited a larger generalization (out-of-sample) error (MAE of 202.50 kcal vs MAE of 183.97 kcal). Figure 3 shows the weights of a conventional linear regression and the elastic net regression with identical predictors. Overall, the elastic net exhibits smaller weights when compared to the linear regression, in all predictors but state chocolate craving, which is attenuated upwards when the model corrects for collinearities. Unexpectedly, the elastic net did not zero out any of the weights despite their high intercorrelations.

As a final step, and considering the low degree of feature selection in the elastic net model, we also conducted another analysis setting *ratio_L1_* to 1.0, which effectively selects an L1 penalty and approximates the stronger feature selection properties of Lasso regression, leading to more sparse models with fewer predictors. All results are shown in Figure 3, main text. The model fit was similar to both the linear regression and the elastic net model with balanced feature selection (in-sample MAE: 165.83 kcal; out-of-sample MAE: 184.65 kcal). The resulting model selected trait chocolate craving, trait impulsivity, stop-signal reaction time, implicit avoidance, peak velocity and total dwell time, as concurrent predictors of chocolate intake.

1. There were two exceptions of participants who performed the web-based tasks already 3 days before the BTT, because their appointments were rescheduled. [↑](#footnote-ref-1)
